# Machine-Learning-Assisted Risk Stratification for Tuberculosis Infection in High-Risk Groups Receiving Immunosuppressive Therapy

**DOI:** 10.3390/diagnostics16213693

**Published:** 2026-11-09

**Authors:** Anna Starshinova, Adilia Sabirova, Olesia Koroteeva, Raul Sharipov, Aleksandr Panteleev, Michail Nazarenko, Nikolay N. Osipov, Dmitry Spelnikov, Dmitry Kudlay

**Affiliations:** 1 Almazov National Research Medical Center, 197341 St. Petersburg, Russia; 2 Faculty of Mathematics and Computer Science, St. Petersburg State University, 199034 St. Petersburg, Russia; 3 Medical Department, Bashkir State Medical University of the Ministry of Health of the Russian Federation, 450000 Ufa, Russia; 4 Medical Department, St. Petersburg State Medical University Named After Academician I.P. Pavlov, 197376 St. Petersburg, Russia; 5 St. Petersburg Research Institute of Phthisiopulmonology, 191036 St. Petersburg, Russia; 6 St. Petersburg State Budgetary Healthcare Institution “City Tuberculosis Dispensary”, 127006 St. Petersburg, Russia; 7 St. Petersburg Department of Steklov Mathematical Institute, 191023 St. Petersburg, Russia; 8 Department of Pharmacology, Institute of Pharmacy, Sechenov University, 119002 Moscow, Russia; 9 FSBI SSC Institute of Immunology FMBA of Russia, 115522 Moscow, Russia; 10 Faculty of Bioengineering and Bioinformatics, Lomonosov Moscow State University, 119991 Moscow, Russia

**Keywords:** tuberculosis, tuberculosis infection, latent tuberculosis infection, immunosuppression, solid-organ transplantation, immunodiagnosis, machine learning, risk stratification

## Abstract

**Background:** Individuals receiving immunosuppressive therapy, solid-organ transplant recipients, household contacts of patients with tuberculosis, and healthcare workers are at increased risk of *Mycobacterium tuberculosis* infection and progression to tuberculosis disease. However, currently available immunological tests cannot reliably distinguish tuberculosis infection from active tuberculosis disease or predict individual progression to disease. We aimed to assess the prevalence of immunological evidence of tuberculosis infection across clinically and epidemiologically distinct risk groups and to explore the potential of machine-learning methods for individualised risk stratification. **Methods:** In this observational study, 544 adults aged 18–75 years were assessed between 2023 and 2026. Participants included patients with pulmonary tuberculosis, tuberculosis-facility healthcare workers, healthcare workers in the general medical network, household and occupational contacts of patients with tuberculosis, patients with rheumatic diseases receiving immunosuppressive therapy, patients undergoing assessment for heart transplantation, heart-transplant recipients, and healthy controls. Participants underwent clinical, epidemiological, radiological, and immunological assessment, including interferon-γ release assays (ELISPOT and QuantiFERON-TB Gold) and the tuberculosis recombinant allergen skin test. Individuals with positive immunological tests underwent additional assessment to exclude active tuberculosis. Exploratory logistic regression and decision-tree models were developed separately for each risk group using demographic and treatment-related variables. Model performance was estimated using repeated stratified 5-fold cross-validation (5 × 10 repeats) with bootstrap optimism correction; discrimination, calibration, sensitivity, specificity, positive and negative predictive values, Cohen’s kappa, and Matthews correlation coefficient were reported with 95% confidence intervals. **Results:** The prevalence of positive immunological tests was highest among heart-transplant recipients (15 [53.6%] of 28), followed by household contacts (12 [41.4%] of 29), patients undergoing pre-transplant assessment (5 [35.7%] of 14), and tuberculosis-facility healthcare workers (21 [22.6%] of 93). Positivity was lower among patients with rheumatic diseases receiving immunosuppressive therapy (11 [13.9%] of 79) and healthcare workers in the general medical network without documented tuberculosis exposure (7 [10.5%] of 68), and was 3 (4.4%) of 69 among healthy controls. Compared with healthy controls, positivity was significantly higher among tuberculosis-facility healthcare workers, household contacts, patients with rheumatic diseases, and pre-transplant and heart-transplant populations. Exploratory machine-learning analyses were performed separately for the two clinically most relevant immunosuppressed subgroups. In patients with rheumatic diseases (*n* = 71; 9 positive, 62 negative), pooled ROC AUC was 0.670 (95% CI 0.520–0.824, participant-level bootstrap); age showed 100% bootstrap sign consistency, whereas bDMARD treatment showed 84.6% and sex 55.0%. In the combined heart transplantation group (*n* = 41; 19 positive, 22 negative), pooled ROC AUC was 0.668 (95% CI 0.522–0.811); tacrolimus and everolimus showed the highest bootstrap sign consistency (100% and 97.6%, respectively); however, because tacrolimus and everolimus are administered predominantly to transplant recipients, these coefficients may partly reflect transplantation status rather than drug-specific effects. Because the heart transplantation group combines pre-transplant candidates and recipients, and because both subgroups had small numbers of events, these associations should be interpreted as exploratory and not as drug-specific causal effects. **Conclusions:** The prevalence of immunological evidence of tuberculosis infection varied substantially between risk groups and was particularly high among household contacts and heart-transplant recipients. The findings suggest that epidemiological exposure and the type of immunosuppressive therapy should be considered jointly when assessing tuberculosis infection risk. Exploratory machine-learning analyses suggested that treatment-related variables may contribute to individualised risk stratification in the combined heart transplantation group, but the models were developed in small, clinically heterogeneous subgroups and were not externally validated. They should therefore be regarded as hypothesis-generating. Prospective multicentre studies with longitudinal follow-up are required to determine whether integrated clinical, epidemiological, immunological, and treatment-related models can predict incident tuberculosis disease and improve targeted preventive strategies.

## 1. Introduction

Currently, several immunological tests are used in the Russian Federation for the detection of tuberculosis infection, including the Mantoux tuberculin skin test with 2 tuberculin units (TU) of purified protein derivative (PPD-L), the tuberculosis recombinant allergen test (Diaskintest^®^), and interferon-γ release assays (IGRAs) [1,2,3,4]. These diagnostic approaches are recommended for use in the Russian Federation and, where applicable, internationally in accordance with World Health Organization (WHO) guidance [5]. Despite the development and introduction of novel immunological assays, the identification of tuberculosis infection remains an important clinical and public health challenge both in the Russian Federation and globally.

Several limitations of currently available immunological tests contribute to this persistent challenge. None of the available assays provides 100% diagnostic accuracy in terms of either sensitivity or specificity. Furthermore, immunological tests alone cannot reliably distinguish tuberculosis infection from tuberculosis disease or accurately predict which individuals with tuberculosis infection will subsequently develop active tuberculosis [4,6,7].

These limitations may result in both underdiagnosis and overdiagnosis of tuberculosis infection owing to false-negative and false-positive test results, respectively. Consequently, preventive treatment may be initiated either too late or unnecessarily in individuals who are unlikely to benefit from such intervention [6,8].

Evidence regarding the diagnostic and prognostic value of immunological markers of tuberculosis infection activity remains inconsistent, and no universally accepted criteria for their interpretation in individuals belonging to high-risk populations have been established [3,9]. Therefore, an integrated assessment combining immunological parameters with the results of tuberculosis infection testing may provide a more informative approach to individual risk stratification and warrants further investigation.

In recent years, increasing attention has been directed towards the application of machine-learning methods to medical data analysis [7]. These approaches can identify complex and potentially non-linear relationships within multidimensional datasets, develop predictive models, and quantify the contribution of individual clinical, epidemiological, and immunological factors to disease risk.

In the present study, we applied exploratory machine-learning methods to assess whether routinely available clinical, epidemiological, and treatment-related variables could contribute to individualised risk stratification for immunological evidence consistent with tuberculosis infection in high-risk populations. The modelled outcome was immunological test positivity, defined as at least one positive result on any of the three immunological tests (ELISPOT, QuantiFERON-TB Gold, or Diaskintest) in the absence of clinical and radiological evidence of active tuberculosis. Our aim was to explore whether integrating these data could improve the identification of individuals with such immunological evidence, including those receiving immunosuppressive therapy.

## 2. Materials and Methods

### 2.1. Study Population and Design

Between 2023 and 2026, 544 participants were enrolled in the study. Of these, 45.5% were men (95% CI 41.23–49.71) and 54.5% were women (95% CI 50.29–58.77). Overall, 51.1% of participants were aged 18–44 years, and the mean age was 41.9 ± 1.35 years.

**Figure 1:**
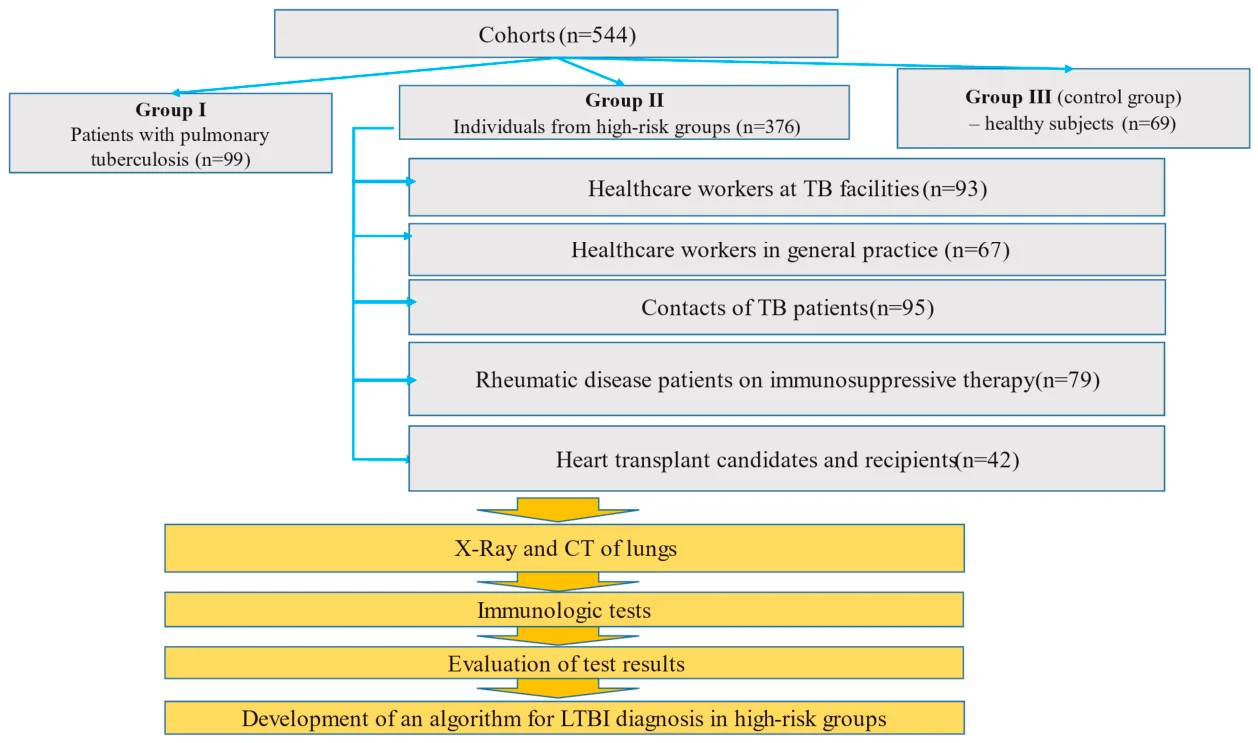
Study design and analytical workflow.

Participants were stratified according to their clinical status, medical history, epidemiological characteristics, and clinical and laboratory findings. The overall study design and distribution of participants across study groups are presented in Figure 1.

Participants were stratified into patients with active pulmonary tuberculosis, individuals from predefined tuberculosis-risk groups, and healthy controls. Participants underwent clinical and epidemiological assessment, chest radiography, and immunological testing using Diaskintest^®^ and interferon-γ release assays. Active tuberculosis was excluded in participants with positive immunological results using clinical, radiological, microbiological, and, where indicated, histopathological assessment. Tuberculosis infection was defined as a positive immunological test result in the absence of evidence of active tuberculosis. Exploratory machine-learning models, including logistic regression and decision-tree analysis, were subsequently used to assess predictors of tuberculosis infection, with particular emphasis on epidemiological exposure and immunosuppressive treatment.

The study cohort was divided into three principal groups. Group 1 comprised patients with pulmonary tuberculosis (*n* = 99). Group 2 comprised individuals at increased risk of tuberculosis (*n* = 376) who had no clinical or radiological evidence of active tuberculosis and no previous history of tuberculosis. Group 2 was further subdivided into five risk categories: health-care workers employed in specialized tuberculosis facilities (*n* = 93); health-care workers in general medical settings (*n* = 68); individuals with documented household or occupational contact with a patient with tuberculosis (*n* = 95); patients with rheumatic diseases receiving immunosuppressive therapy (*n* = 79); and patients undergoing assessment for heart transplantation or recipients of a transplanted heart (*n* = 42).

Group 3 was the control group and comprised apparently healthy individuals (*n* = 69) without documented contact with a patient with tuberculosis, clinical or radiological evidence of tuberculosis infection, acute or chronic disease, or a previous history of tuberculosis.

The study population therefore included individuals representing a spectrum of tuberculosis risk, ranging from patients with confirmed pulmonary tuberculosis to individuals with occupational or epidemiological exposure, patients receiving immunosuppressive therapy, patients undergoing or having undergone heart transplantation, and healthy controls.

#### 2.1.1. Eligibility Criteria

Eligible participants were men and women aged 18–75 years, inclusive, who belonged to one of the predefined study populations. These included patients with pulmonary tuberculosis; health-care workers employed in general medical facilities who had no documented contact with patients with tuberculosis; health-care workers employed in specialized tuberculosis facilities; individuals with a documented household or occupational history of contact with a patient with tuberculosis; patients with rheumatic diseases receiving immunosuppressive therapy; patients on the waiting list for heart transplantation or undergoing assessment for inclusion on the waiting list; heart transplant recipients; and apparently healthy individuals without documented contact with patients with tuberculosis, clinical or radiological evidence of tuberculosis infection, acute disease, or exacerbation of chronic disease.

*The inclusion criteria were therefore:*

- Age 18–75 years, irrespective of sex;
- Employment in general medical hospitals or outpatient facilities without routine involvement in the care of patients with tuberculosis;
- Employment in specialised tuberculosis facilities;
- Previous household or occupational contact with a patient with bacteriologically confirmed tuberculosis;
- Rheumatic disease requiring immunosuppressive therapy;
- Cardiac disease requiring pre-transplant assessment or heart transplantation, including patients awaiting transplantation and heart transplant recipients;
- An apparently healthy status, with no documented contact with patients with tuberculosis, no characteristic radiological abnormalities or clinical symptoms suggestive of tuberculosis, and no acute illness or exacerbation of chronic disease.

#### 2.1.2. Exclusion Criteria

Participants were excluded if they were younger than 18 years or older than 75 years; had disseminated or generalized tuberculosis; had disease caused by non-tuberculous mycobacteria; had a primary immunodeficiency; had HIV infection; had a malignant neoplasm; were receiving haemodialysis; had an exacerbation of a chronic disease; were pregnant or breastfeeding; or had a previous history of active tuberculosis.

Individuals who did not provide written informed consent were also excluded from participation.

#### 2.1.3. Withdrawal Criteria

Participants were withdrawn from the study if they subsequently withdrew their informed consent, if the protocol for immunological assessment was violated, or if a clinical condition was identified during the study that was inconsistent with the predefined eligibility criteria.

#### 2.1.4. Characteristics of the Tuberculosis Group

Among patients in Group 1, the most common clinical forms of pulmonary tuberculosis were infiltrative tuberculosis (33.3%) and fibrocavernous pulmonary tuberculosis (29.3%). Overall, 48.5% of patients included in the tuberculosis group had bacteriologically confirmed disease with mycobacterial excretion, whereas drug resistance of Mycobacterium tuberculosis was identified in 37.5% of cases.

#### 2.1.5. Characteristics of Occupationally Exposed Participants

All health-care workers employed in specialized tuberculosis facilities (*n* = 93) had occupational contact with patients with tuberculosis.

All health-care workers in general medical settings (*n* = 68) were employed in general medical facilities, excluding institutions providing care for patients with infectious diseases. None had a documented history of contact with a patient with tuberculosis.

#### 2.1.6. Characteristics of Tuberculosis Contacts

All individuals with documented contact with tuberculosis (*n* = 95) were followed up in the outpatient service of a specialized tuberculosis facility within the IV-A dispensary surveillance category, as individuals exposed to a source of tuberculosis infection.

Of these participants, 30.5% (29/95) had household contact and 69.5% (66/95) had occupational contact with a patient with tuberculosis. In 89.5% (85/95) of cases, the source patient had bacteriologically confirmed tuberculosis with mycobacterial excretion. 

#### 2.1.7. Patients with Rheumatic Diseases Receiving Immunosuppressive Therapy

Among patients with rheumatic diseases, documented contact with a patient with tuberculosis was identified in only one participant (1.3%), and this contact was occupational.

Overall, 83.5% (66/79) of patients with rheumatic diseases received genetically engineered biological therapies. This subgroup therefore represented a clinically relevant population at increased risk of tuberculosis associated with immunosuppressive treatment.

#### 2.1.8. Patients Undergoing Heart Transplantation and Heart Transplant Recipients

The heart transplantation subgroup (*n* = 42) comprised patients undergoing pre-transplant assessment or awaiting heart transplantation (*n* = 14) and heart transplant recipients (*n* = 28).

None of the 42 participants in this subgroup had a documented history of contact with a patient with tuberculosis.

#### 2.1.9. Control Group

The control group consisted of apparently healthy individuals without a documented history of contact with patients with tuberculosis, clinical symptoms or radiological findings suggestive of tuberculosis infection, acute disease, or exacerbation of chronic disease. Individuals with a previous history of tuberculosis were also excluded from the control group.

#### 2.1.10. Study Groups

The final study population was therefore structured to enable comparison across distinct levels and mechanisms of tuberculosis risk: confirmed pulmonary tuberculosis; occupational exposure in specialised tuberculosis facilities; employment in general medical settings without documented tuberculosis exposure; household or occupational contact with tuberculosis; immunosuppression associated with treatment of rheumatic diseases; the peri-transplant and post-transplant setting; and healthy individuals without recognised tuberculosis risk factors.

This stratification was subsequently used for the analysis of immunological parameters and the development of exploratory machine-learning models for the identification of individuals with immunological evidence consistent with tuberculosis infection.

Data on tuberculosis exposure among participants in the high-risk groups are presented in Table 1.

All health-care workers employed in specialized tuberculosis facilities (*n* = 93) had occupational exposure to patients with tuberculosis, including patients with bacteriologically confirmed disease and patients with multidrug-resistant (MDR) and extensively drug-resistant (XDR) tuberculosis.

All health-care workers employed in general medical facilities (*n* = 68) worked in general hospitals or outpatient medical facilities, excluding institutions providing care for patients with infectious diseases. None had a documented history of contact with a patient with tuberculosis.

All individuals with documented tuberculosis exposure (*n* = 95) were followed up in the outpatient service of a specialized tuberculosis facility within the IV-A dispensary surveillance category for individuals exposed to a source of tuberculosis infection. All participants in this subgroup underwent additional clinical and radiological assessment to exclude active tuberculosis. Household exposure was reported in 30.5% (29/95) of participants, whereas 69.5% (66/95) had occupational exposure. In 89.5% (85/95) of cases, the source patient had bacteriologically confirmed tuberculosis.

Among patients with rheumatic diseases receiving immunosuppressive therapy, documented contact with a patient with tuberculosis was identified in only 1.3% (1/79) of participants and was occupational in nature.

None of the patients undergoing pre-transplant assessment or awaiting heart transplantation, or heart transplant recipients (*n* = 42), had a documented history of contact with a patient with tuberculosis.

All patients with rheumatic diseases were regularly followed up by a rheumatologist to monitor disease activity and optimise maintenance therapy. The immunosuppressive agents administered to these patients are presented in Table 2. Overall, 83.5% (66/79) of patients received genetically engineered biological therapies.

The subgroup of patients undergoing heart transplantation and heart transplant recipients (*n* = 42) comprised 14 patients undergoing pre-transplant assessment and 28 heart transplant recipients.

Patients undergoing pre-transplant assessment (*n* = 14) had clinical and instrumental evidence of chronic heart failure and underwent comprehensive diagnostic evaluation to determine their eligibility for heart transplantation. The clinical and demographic characteristics of patients undergoing pre-transplant assessment are presented in Table 3.

All patients with chronic heart failure associated with reduced left ventricular ejection fraction received guideline-directed quadruple therapy comprising an angiotensin receptor–neprilysin inhibitor, a β-blocker, a mineralocorticoid receptor antagonist, and a sodium–glucose cotransporter-2 (SGLT2) inhibitor.

Among the heart transplant recipient subgroup (*n* = 28), all participants were undergoing lifelong multidisciplinary follow-up involving transplant physicians, cardiologists, cardiac surgeons, clinical pharmacologists, and other specialists to prevent and detect both cellular and antibody-mediated graft rejection.

The immunosuppressive agents included in the treatment regimens of heart transplant recipients are presented in Table 4.

All participants subsequently underwent a comprehensive clinical and laboratory assessment, including an immunological evaluation. When an immunological test was positive, additional diagnostic investigations were performed to exclude active tuberculosis in accordance with current national diagnostic standards and clinical guidelines.

The diagnostic assessment included clinical evaluation and appropriate radiological and microbiological investigations, as indicated, to distinguish tuberculosis infection from active tuberculosis disease before inclusion in the risk-stratification analysis.

### 2.2. Study Methods

#### 2.2.1. Clinical, Epidemiological and Radiological Assessment

The assessment included a detailed medical history, the participant’s general clinical condition, the presence or absence of symptoms in patients with tuberculosis, the clinical form of tuberculosis, and the drug-resistance profile of the causative organism. Information on previous contact with a patient with active tuberculosis was recorded, including the type of exposure and whether the index patient had bacteriologically confirmed tuberculosis with mycobacterial excretion. For health-care workers, occupational setting was documented. For participants in the high-risk groups, the underlying medical condition and the type of immunosuppressive therapy were recorded. The results of clinical, laboratory, microbiological, and radiological investigations were also included in the analysis.

All participants in the high-risk and healthy control groups underwent chest radiography to exclude radiological evidence of tuberculosis, together with immunological testing for tuberculosis infection. The immunological assessment included interferon-γ release assays (IGRAs), specifically ELISPOT and QuantiFERON-TB Gold, as well as the Diaskintest^®^ assay.

Radiological findings were assessed according to the anatomical distribution and characteristics of pulmonary abnormalities, including unilateral or bilateral involvement, solitary or multiple lesions, lesion size, and the presence or absence of intrathoracic lymphadenopathy.

#### 2.2.2. Diagnostic Criteria

A diagnosis of pulmonary tuberculosis was established on the basis of compatible clinical manifestations, characteristic radiological findings, and positive microbiological or histopathological evidence. Microbiological confirmation was based on the detection of *Mycobacterium tuberculosis* in sputum specimens using bacteriological methods. Histopathological confirmation was based on the identification of epithelioid-cell granulomas with areas of caseous necrosis and acid-fast bacilli in lung tissue.

Latent tuberculosis infection (LTBI) was defined as at least one positive result on any of the three immunological tests (ELISPOT, QuantiFERON-TB Gold, or Diaskintest) in the absence of clinical symptoms and clinical or radiological evidence of tuberculosis disease [5].

#### 2.2.3. Statistical Analysis

Statistical analyses and graphical data visualisation were performed using Microsoft Excel 2019 (Microsoft, Redmond, WA, USA), STATISTICA version 8.0 (StatSoft Inc., Tulsa, OK, USA), GraphPad Prism version 5.0 (GraphPad Software, Reston, VA, USA), and Python 3.10, using the pandas, SciPy, and scikit-learn libraries.

Continuous variables were assessed using standard descriptive statistical methods. The distribution of continuous variables was evaluated using the Shapiro–Wilk test for groups with fewer than 50 participants and the Kolmogorov–Smirnov test for groups with more than 50 participants.

Continuous variables were summarized as the arithmetic mean (mean), standard deviation (SD), and 95% confidence interval (CI) for the mean. Categorical variables were reported as absolute numbers (n/N) and percentages (%).

Confidence intervals for continuous variables, including age, were calculated using Student’s *t* distribution. For binomial proportions, including sex, type of tuberculosis exposure, and immunological test results, confidence intervals were initially calculated using the Wald normal approximation. When the resulting lower bound was below 0% or the upper bound exceeded 100%, a corrected Wald method or Wilson score interval was used. All confidence intervals were calculated at the 95% confidence level.

Confidence intervals were presented graphically using box-and-whisker plots generated in Microsoft Excel 2019.

The prevalence of LTBI in the high-risk and control groups was estimated as the proportion of participants with positive immunological test results. Statistical significance was defined as a two-sided *p* value of less than 0.05.

Differences in the results of immunological testing between the study groups were assessed using Pearson’s χ^2^ test. Where appropriate, categorical variables were compared using contingency-table methods.

Flow-cytometric data were processed using Kaluza™ version 2.0 software (Beckman Coulter, Brea, CA, USA).

Categorical variables were compared using Pearson’s χ^2^ test when its assumptions were satisfied. For comparisons involving small expected cell frequencies (less than five), Fisher’s exact test was considered the more appropriate statistical method. Pairwise comparisons of LTBI prevalence between the study groups were regarded as exploratory. Given the multiple comparisons performed, unadjusted *p* values were interpreted cautiously, particularly when approaching the conventional significance threshold of 0.05. Effect sizes, 95% confidence intervals, and clinical relevance were considered alongside statistical significance.

### 2.3. Machine-Learning Methods

A binary classification framework was used to develop exploratory models, with immunological evidence consistent with tuberculosis infection as the target outcome. Class 1 represented participants with at least one positive immunological test (ELISPOT, QuantiFERON-TB Gold, or Diaskintest) in the absence of clinical and radiological evidence of active tuberculosis. Class 0 represented participants without such evidence. Model development and evaluation were performed separately for each predefined high-risk group.

The predictor variables included sex and age, tuberculosis contact variables (documented contact with a patient with tuberculosis, type of contact, and bacteriological status of the source patient). Age was analysed as a continuous variable in the final models. This choice was informed by a pre-specified sensitivity analysis comparing continuous age, ordinal age category (six categories), and one-hot encoding of age categories; one-hot encoding performed substantially worse (ΔAUC = −0.08 in the rheumatic disease group and −0.05 in the heart transplantation group), consistent with a reduction in events per variable. For patients with rheumatic diseases, a binary predictor indicating treatment with biological disease-modifying antirheumatic drugs (bDMARDs) was included. For patients in the heart transplantation group, treatment-related binary predictors included tacrolimus, everolimus, mycophenolate mofetil, glucocorticoids, and ciclosporin, together with sex and age.

Missing data were handled as follows: Observations with missing values were excluded from the corresponding analysis. No imputation was performed. The number of excluded observations and the resulting sample sizes are reported for each group.

Two classification algorithms were evaluated to provide complementary perspectives: L2-regularised logistic regression as a parametric, interpretable reference model, and a decision tree classifier as a non-parametric approach capable of capturing non-linear relationships and interactions. These two algorithms were selected because their interpretability—regression coefficients for logistic regression and feature importance for decision trees—is essential for exploratory hypothesis generation in small clinical datasets. Model development and reporting followed the TRIPOD+AI and PROBAST+AI recommendations for regression and machine-learning models.

Both models used class weight = ‘balanced’ to account for class imbalance. Continuous predictors were standardised to mean zero and standard deviation one inside a pipeline, so that reported coefficients correspond to a one-standard-deviation change in the predictor. The classification threshold was fixed at 0.5. The random seed was pre-specified as 42 for all analyses to ensure reproducibility.

Logistic regression was fitted with L2 regularisation to reduce overfitting; the inverse regularisation strength C was tuned over 11 values (0.001, 0.003, 0.01, 0.03, 0.1, 0.3, 1, 3, 10, 30, 100). The final selected value was C = 0.03 for the rheumatic disease group and C = 0.3 for the heart transplantation group. Coefficients are reported in the original scale of the predictors together with the numerical intercept.

Decision trees were fitted with the following hyperparameters tuned by nested stratified cross-validation: maximum depth ∈ {2, 3, 4, 5}, minimum samples per split ∈ {2, 3, 5}, minimum samples per leaf ∈ {1, 2, 3}, and split criterion ∈ {Gini, entropy}. The final selected configurations were entropy, max_depth = 2, min_samples_leaf = 1, min_samples_split = 2 for the rheumatic disease group; and Gini, max_depth = 2, min_samples_leaf = 2, min_samples_split = 2 for the heart transplantation group. Feature importance was based on impurity reduction across the tree.

Model development was performed in Python 3.9.12 using scikit-learn 1.0.2; NumPy and pandas versions were 1.21 and 1.3, respectively.

### 2.4. Model Performance and Interpretability

Because a single random training–test split produces unstable estimates in small datasets, model performance was estimated using repeated stratified 5-fold cross-validation with 10 repeats (5 × 10), producing pooled out-of-fold predictions. Hyperparameters were tuned within the cross-validation loop using nested stratified 5-fold cross-validation; the test folds were not used during model development or hyperparameter selection. The final reported AUC values are based on pooled out-of-fold predictions across all 50 folds, which reduces the dependence of the results on any single random split.

Discrimination was assessed using the area under the receiver operating characteristic curve (ROC AUC) and the area under the precision–recall curve (PR-AUC). Additional metrics included sensitivity, specificity, positive predictive value (PPV), negative predictive value (NPV), Cohen’s kappa, and Matthews correlation coefficient (MCC). Calibration was assessed using the Brier score, calibration intercept, and calibration slope. Bootstrap optimism correction (1000 resamples) was applied to estimate the residual optimism of ROC AUC. 95% confidence intervals for all metrics were obtained using bootstrap resampling at the participant level (1000 resamples), preserving the clustering structure induced by repeated cross-validation. For each bootstrap iteration, participants were resampled with replacement, and all out-of-fold predictions from the same participant were retained together. This approach avoids pseudo-replication. Accuracy and F1 score were not reported because they are less informative than the other metrics under class imbalance and were not part of the prespecified analysis plan.

The final logistic regression model for each analysed subgroup is reported in the Results section (Section 3.3) in the original scale of the predictors, with numerical intercepts. Age is expressed as a continuous variable in years; sex is coded as 0 for female and 1 for male; and drug treatment variables (bDMARD, tacrolimus, everolimus, mycophenolate mofetil, glucocorticoids, ciclosporin) are coded as 1 for presence and 0 for absence of the drug. Regression coefficients quantify the change in log-odds associated with a one-unit change in the corresponding predictor (for continuous or categorical predictors) or with the presence of the corresponding binary predictor. In these equations, logit(p) is the natural logarithm of the odds of immunological evidence consistent with tuberculosis infection, transforming a probability *p* (0 < *p* < 1) into log-odds.

For logistic regression, the sign and magnitude of each coefficient were interpreted as exploratory associations with the log-odds of immunological evidence consistent with tuberculosis infection. A positive coefficient indicates increased model-estimated odds; a negative coefficient indicates decreased odds. Because of the small number of events, coefficients should not be interpreted as causal effects. To assess the stability of the estimated coefficients, bootstrap resampling was performed with 1000 resamples. For each predictor, the mean coefficient, its 2.5th and 97.5th percentiles, and the proportion of resamples in which the sign of the coefficient matched the sign of the mean estimate (sign consistency) were recorded and are reported in the Results section.

For decision trees, feature importance was based on impurity reduction across the tree. Bootstrap resampling with 1000 resamples was used to assess the stability of feature-importance values; for each predictor, the mean importance, its 95% confidence interval, and the proportion of resamples in which the importance was non-zero were recorded and are reported in the Results section. Feature importance values should not be interpreted as causal effects or as a definitive hierarchy of risk factors.

### 2.5. Methodological Considerations

The machine-learning component was exploratory and hypothesis-generating. The number of events was small in both analysed subgroups (9 of 71 in the rheumatic disease group and 19 of 41 in the heart transplantation group), which limits the precision of the estimates and the complexity of the models that can be reliably fitted. External validation was not performed. The modelled outcome was immunological test positivity rather than incident active tuberculosis; accordingly, the models should be described as models of immunological evidence consistent with tuberculosis infection, not as validated models predicting progression to active tuberculosis.

## 3. Results

### 3.1. Immunological Testing in High-Risk Groups

The results of immunological testing among participants in the high-risk groups are presented in Table 5.

Positive immunological test results were more frequent in all predefined high-risk groups than in healthy controls, among whom 3 (4.4%) of 69 participants had a positive result.

Among health-care workers employed in general medical facilities, immunological tests were positive in 7 (10.5%) of 68 participants. Although this proportion was higher than that observed in healthy controls (3 [4.4%] of 69), the difference was not statistically significant (*p* = 0.174).

By contrast, statistically significant differences compared with the healthy control group were observed in the other high-risk populations. Positive immunological results were detected in 21 (22.6%) of 93 health-care workers employed in specialised tuberculosis facilities (*p* = 0.002), 12 (41.4%) of 29 individuals with household exposure to tuberculosis (*p* = 0.001), and 14 (21.2%) of 66 individuals with occupational exposure (*p* = 0.004).

Among patients with rheumatic diseases receiving immunosuppressive therapy, 11 (13.9%) of 79 participants had positive immunological test results, which was significantly higher than the prevalence observed in healthy controls (*p* = 0.048).

Positive immunological results were also frequent among patients undergoing pre-transplant assessment and heart transplant recipients: 5 (35.7%) of 14 and 15 (53.6%) of 28 participants, respectively. Both groups showed significantly higher proportions of positive results than healthy controls (*p* = 0.001 for each comparison).

Thus, the highest frequency of positive immunological test results among the high-risk groups was observed in heart transplant recipients (53.6%), followed by individuals with household exposure to tuberculosis (41.4%) and patients undergoing pre-transplant assessment (35.7%). In contrast, the frequency among health-care workers in general medical facilities (10.5%) was relatively close to that observed in the healthy control group (4.4%), and the difference did not reach statistical significance.

Comparative analyses of immunological test results across the predefined high-risk groups are presented in Table 6.

### 3.2. Statistically Significant Difference

The original table reports *n* = 93 for the heart transplant recipient group in this comparison; this appears inconsistent with the study population and should be verified against the source dataset.

Positive immunological test results were observed in 10.5% (7/67) of health-care workers employed in general medical facilities without documented tuberculosis exposure. This proportion was significantly lower than that observed among individuals with household exposure (41.4% [12/29]; *p* = 0.001) and health-care workers employed in specialised tuberculosis facilities (22.6% [21/93]; *p* = 0.047). The difference compared with individuals with occupational exposure was not statistically significant (21.2% [14/66]; *p* = 0.089).

Among health-care workers in specialized tuberculosis facilities, the frequency of positive immunological results was significantly lower than that observed among individuals with household exposure (22.6% vs. 41.4%; *p* = 0.047), but did not differ significantly from that among individuals with occupational exposure (22.6% vs. 21.2%; *p* = 0.838).

A statistically significant difference was also observed between individuals with household and occupational tuberculosis exposure, with positive immunological results being more frequent in the household-exposure group (41.4% vs. 21.2%; *p* = 0.043).

No statistically significant difference was observed between patients undergoing pre-transplant assessment and heart transplant recipients (35.7% vs. 53.6%; *p* = 0.275). By contrast, the frequency of positive immunological results was significantly higher among heart transplant recipients than among patients with rheumatic diseases receiving immunosuppressive therapy (53.6% vs. 13.9%; *p* = 0.001).

The relatively high frequency of positive immunological test results among health-care workers in general medical facilities, despite the absence of documented tuberculosis exposure, might indicate the presence of previously unrecognised exposure or other epidemiological or immunological factors. However, the study design does not allow undocumented exposure to be established as the cause of these findings. This observation warrants further investigation using longitudinal studies with more detailed assessment of occupational, household, and community exposure to tuberculosis.

Individuals with household tuberculosis exposure had positive immunological test results in 41.4% (12/29) of cases, which was significantly higher than the proportion observed among individuals with occupational exposure (21.2% [14/66]; *p* = 0.043) and health-care workers employed in specialised tuberculosis facilities (22.6% [21/93]; *p* = 0.047). No statistically significant difference was observed between individuals with occupational exposure and health-care workers in specialised tuberculosis facilities (*p* = 0.838).

Positive immunological test results were observed in 35.7% (5/14) of patients undergoing pre-transplant assessment and 53.6% (15/28) of heart transplant recipients. The difference between these groups was not statistically significant (*p* = 0.275).

By contrast, a significant difference was observed when immunological test results in patients with rheumatic diseases receiving immunosuppressive therapy were compared with those in heart transplant recipients: 13.9% (11/79) versus 53.6% (15/28), respectively (*p* = 0.001). This finding is consistent with a substantially greater degree of immunosuppression among heart transplant recipients and may reflect differences in the intensity, duration, and type of immunosuppressive treatment between these populations.

**Figure 2:**
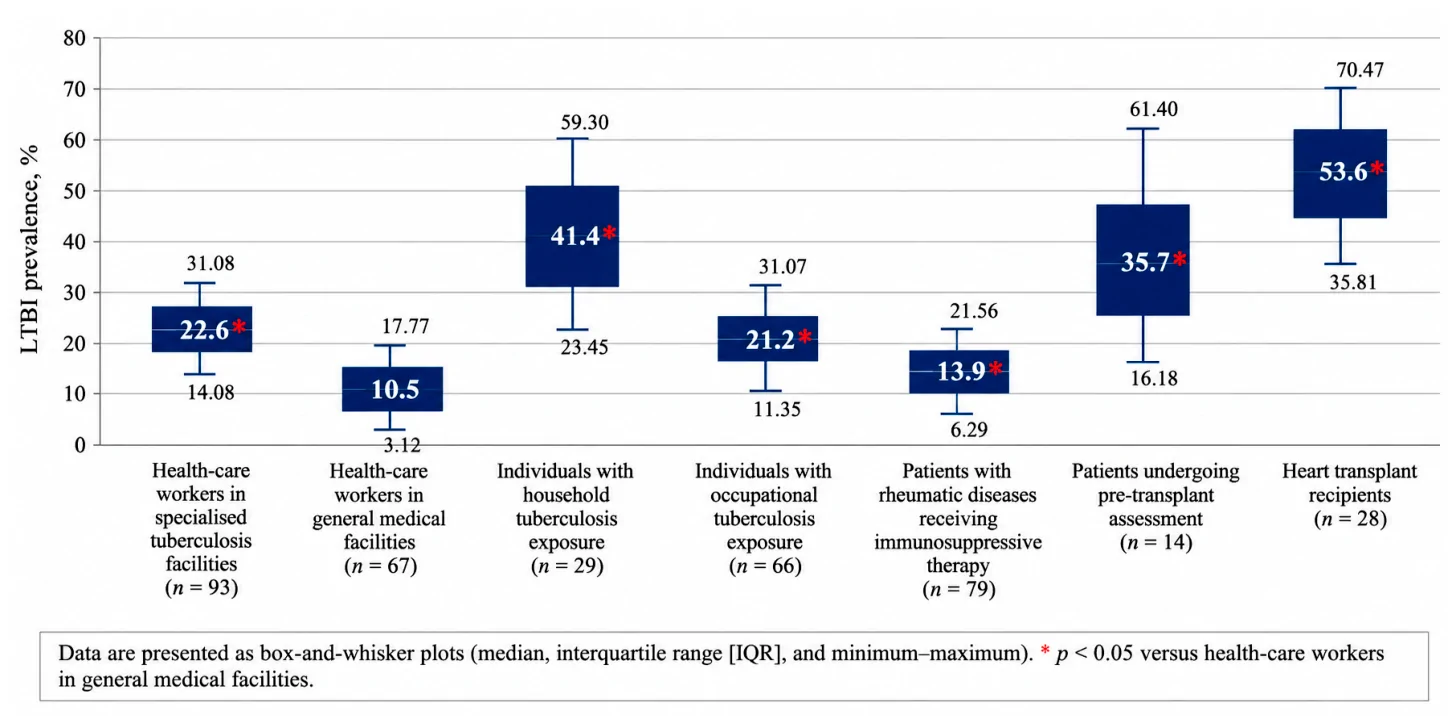
Prevalence of tuberculosis infection in high-risk groups (%).

Because positive immunological test results in individuals from the high-risk groups were identified in the absence of clinical or radiological evidence of tuberculosis disease, these findings were considered consistent with an immunological response to *Mycobacterium tuberculosis* and were used to estimate the prevalence of tuberculosis infection (TBI) in the respective study groups. The prevalence of TBI across the high-risk groups is presented in Figure 2.

The highest prevalence of TBI was observed among heart transplant recipients (53.6%), exceeding that observed in patients undergoing pre-transplant assessment (35.7%). The proportion of positive immunological test results among individuals with household exposure (41.4%) was approximately twice that observed among individuals with occupational exposure (21.2%) and health-care workers employed in specialised tuberculosis facilities (22.6%).

Among patients with rheumatic diseases receiving immunosuppressive therapy, TBI was identified in 13.9% of participants, approximately four times less frequently than among heart transplant recipients (53.6%). The lowest prevalence of TBI among the high-risk groups was observed in health-care workers employed in general medical facilities without documented tuberculosis exposure (10.5%). Nevertheless, this proportion was higher than that observed in the healthy control group (4.4%).

Overall, these findings indicate substantial heterogeneity in the prevalence of immunological evidence of tuberculosis infection across different risk populations. The highest prevalence was observed in heart transplant recipients and individuals with household exposure to tuberculosis, whereas substantially lower proportions were observed among health-care workers without documented exposure and healthy controls.

### 3.3. Exploratory Machine-Learning Analyses

Exploratory machine-learning analyses were performed separately for the two clinically most relevant immunosuppressed subgroups: patients with rheumatic diseases and patients in the heart transplantation group (combining pre-transplant candidates and heart transplant recipients). Results for the other subgroups were not interpretable because of the very small number of events and are not presented.

#### 3.3.1. Patients with Rheumatic Diseases

After exclusion of 8 observations with missing bDMARD data, the analysis included 71 participants (9 with immunological evidence consistent with tuberculosis infection and 62 without). The final predictor set comprised continuous age (in years), sex, and bDMARD treatment. Age was analysed as a continuous variable, based on a pre-specified sensitivity analysis that compared continuous age, ordinal age category (six categories), and one-hot encoding of age categories. One-hot encoding performed substantially worse (ΔAUC = −0.08) because of the reduction in events per variable, whereas continuous age performed at least as well as the ordinal representation while avoiding the assumption of a linear trend across categories.

The logistic regression equation in the original scale in patients with rheumatic diseases of the predictors was:

logit(p) = −1.9210 + 0.0667 × Age (years) − 0.1219 × Sex − 1.2973 × bDMARD treatment

Pooled ROC AUC was 0.670 (95% CI 0.520–0.824, participant-level bootstrap); PR-AUC was 0.207 (95% CI 0.083–0.393). Bootstrap optimism correction produced a corrected AUC of 0.652.

Bootstrap stability analysis (500 resamples) showed the following sign consistency for regression coefficients: age 100%, bDMARD treatment 84.6%, sex 55.0%.

The decision-tree model (entropy, max_depth = 2, min_samples_leaf = 1, min_samples_split = 2) identified age as the only important feature (importance 1.00; bootstrap mean 0.833, non-zero frequency 99.8%). Sex and bDMARD treatment had zero importance in the final tree.

#### 3.3.2. Heart Transplantation Group

Two observations with missing age were excluded, leaving 41 participants (19 with immunological evidence consistent with tuberculosis infection and 22 without). The group combined 14 pre-transplant candidates and 27 recipients in the analysis dataset. The final predictor set comprised continuous age, sex, tacrolimus, everolimus, mycophenolate mofetil, glucocorticoids, and ciclosporin.

The final logistic regression equation in the original scale in the heart transplantation group of the predictors was:

logit(p) = −0.3893 + 0.0107 × Age (years) − 0.6900 × Sex + 1.1691 × Tacrolimus + 0.7506 × Everolimus + 0.1008 × Mycophenolate mofetil − 1.0282 × Glucocorticoids − 1.7896 × Ciclosporin

Pooled ROC AUC was 0.668 (95% CI 0.522–0.811, participant-level bootstrap); PR-AUC was 0.616 (95% CI 0.420–0.827). Bootstrap optimism correction produced a corrected AUC of 0.644.

Bootstrap stability analysis (500 resamples) showed the following sign consistency for regression coefficients: tacrolimus 100%, everolimus 97.6%, glucocorticoids 94.0%, sex 93.0%, ciclosporin 88.8%, age 72.2%, and mycophenolate mofetil 55.2%. Because tacrolimus and everolimus are administered predominantly to post-transplant recipients, the coefficients for these drugs cannot be disentangled from transplantation status in the present analysis.

The decision-tree model (Gini, max_depth = 2, min_samples_leaf = 2, min_samples_split = 2) identified everolimus (importance 0.706) and age (0.294) as the most important features. Bootstrap analysis showed non-zero importance frequencies of 80.0% for age, 56.8% for tacrolimus, 43.2% for glucocorticoids, and 37.0% for everolimus. The remaining drug variables had non-zero frequencies below 10%.

#### 3.3.3. Summary of Model Performance

A summary of the model performance metrics for both subgroups is presented in Table 7. All metrics are reported with 95% confidence intervals obtained using bootstrap resampling at the participant level, which preserves the clustering structure induced by repeated cross-validation. For each bootstrap iteration, participants were resampled with replacement, and all out-of-fold predictions from the same participant were retained together. This approach avoids pseudo-replication.

#### 3.3.4. Interpretation of Model Performance

In the heart transplantation group (group 6), the model demonstrated modest discriminative ability (ROC AUC 0.668, 95% CI 0.522–0.811), with sensitivity 0.579 (95% CI 0.356–0.781) and specificity 0.764 (95% CI 0.596–0.905). The PR-AUC of 0.616 (95% CI 0.420–0.827) substantially exceeded the baseline prevalence of 0.463 (19 of 41), indicating that the model provided meaningful enrichment over chance. PPV was 0.679 (95% CI 0.451–0.876), indicating that approximately two-thirds of positive predictions were correct; NPV was 0.677 (95% CI 0.498–0.857). Agreement beyond chance was moderate (Cohen’s kappa 0.346, 95% CI 0.080–0.588; MCC 0.349, 95% CI 0.082–0.599). Bootstrap stability analysis confirmed high sign consistency for tacrolimus (100%) and everolimus (97.6%); however, because tacrolimus and everolimus are administered predominantly to post-transplant recipients, these coefficients may partly reflect transplantation status rather than drug-specific effects. Calibration was poor, with a Brier score of 0.241 (95% CI 0.200–0.284) and a calibration slope of 0.112, indicating substantial miscalibration and unsuitability for absolute risk estimation.

In the rheumatic disease group (group 5), the model showed modest discriminative ability (ROC AUC 0.670, 95% CI 0.520–0.824), with sensitivity 0.522 (95% CI 0.250–0.817) and specificity 0.716 (95% CI 0.613–0.814). The relatively high NPV (0.912, 95% CI 0.830–0.977) reflects the low prevalence of the outcome (9 of 71; baseline NPV without any model = 0.873) rather than reliable rule-out performance; the model offers only marginal improvement over a naive all-negative strategy, and PPV was 0.211 (95% CI 0.071–0.384), indicating difficulty in identifying positive cases. Agreement beyond chance was low (Cohen’s kappa 0.146, 95% CI −0.021 to 0.340; MCC 0.171, 95% CI −0.028 to 0.382), with confidence intervals that included zero. Bootstrap stability analysis showed 100% sign consistency for age but lower stability for bDMARD treatment (84.6%) and sex (55.0%), indicating that the direction of the association for these variables is not firmly established. Calibration slope was low (0.082), indicating substantial miscalibration.

Pooled ROC AUC values were modest (0.670 and 0.668), and 95% confidence intervals were relatively wide because participant-level bootstrap was used to account for the clustering structure of repeated cross-validation. Because of the small number of events (9 and 19, respectively), the clinical heterogeneity within the heart transplantation group (which combines pre-transplant candidates and recipients), and the absence of external validation, these findings should be interpreted as hypothesis-generating and not as validated prediction of individual risk.

### 3.4. Summary of the Main Findings

The prevalence of immunological evidence consistent with TBI among health-care workers in general medical facilities was slightly higher than that observed in healthy controls (10.5% vs. 4.4%), although this difference was not statistically significant.

Among health-care workers employed in specialised tuberculosis facilities, the prevalence of positive immunological test results was significantly higher than that observed among health-care workers in general medical facilities (22.6% vs. 10.5%; *p* = 0.047), but was similar to that observed among individuals with occupational exposure to tuberculosis (21.2%; *p* = 0.838).

Among individuals with household exposure to tuberculosis, positive immunological test results were detected in 41.4% of participants. This proportion was approximately twice that observed among individuals with occupational exposure (21.2%; *p* = 0.043) and was significantly higher than that among health-care workers in general medical facilities (10.5%; *p* = 0.001).

Heart transplant recipients had a substantially higher prevalence of positive immunological test results than patients with rheumatic diseases receiving immunosuppressive therapy (53.6% vs. 13.9%; *p* = 0.001), despite both groups receiving long-term immunosuppressive treatment. Within the heart transplant recipient subgroup, tacrolimus and everolimus treatment were among the most important treatment-related predictors in the logistic regression model, suggesting differential associations between individual immunosuppressive regimens and the probability of TBI.

The reported increase in the prevalence of positive immunological test results among patients with rheumatic diseases receiving immunosuppressive therapy, from 13.9% to 16.7% in the corresponding analysis, further emphasises the need for careful surveillance of this high-risk population. However, this finding should be interpreted cautiously unless the 16.7% estimate derives from a prespecified longitudinal analysis, as the primary cross-sectional estimate for this subgroup was 13.9%.

Overall, immunological testing provides an objective tool for identifying individuals with an immune response consistent with tuberculosis infection among populations at increased risk. Integration of immunological testing with clinical, epidemiological, treatment-related, and machine-learning-derived predictors may facilitate individualised risk stratification and targeted clinical surveillance. In patients receiving immunosuppressive therapy, such an integrated approach could support the identification of individuals requiring more detailed assessment for tuberculosis infection and closer follow-up.

In exploratory machine-learning analyses, pooled ROC AUC was 0.670 (95% CI 0.520–0.824) for the rheumatic disease group and 0.668 (95% CI 0.522–0.811) for the heart transplantation group. Bootstrap analyses showed high sign consistency for age in the rheumatic disease group (100%) and for tacrolimus, everolimus, and glucocorticoids in the heart transplantation group (100%, 97.6%, and 94.0%, respectively). In the rheumatic disease group, the coefficient for bDMARD treatment was negative (β = −1.30) but with lower stability (sign consistency 84.6%).

The machine-learning component should be regarded as exploratory and hypothesis-generating. External validation in larger, multicentre cohorts is required before any of the identified associations can be used to guide individualised risk stratification or preventive treatment decisions.

## 4. Discussion

In this study, we evaluated the frequency of immunological evidence of *Mycobacterium tuberculosis* infection across several clinically and epidemiologically distinct risk groups and explored whether routinely available demographic, epidemiological, and treatment-related variables could contribute to individualized risk stratification using exploratory machine-learning approaches. The principal finding was the marked heterogeneity in the prevalence of positive immunological tests between risk groups. The highest proportion was observed among heart-transplant recipients (53.6%), followed by individuals with household exposure to tuberculosis (41.4%), patients undergoing pre-transplant assessment (35.7%), and tuberculosis-exposed healthcare workers (22.6%). By contrast, positivity was substantially lower among patients with rheumatic diseases receiving immunosuppressive treatment (13.9%) and healthcare workers in the general medical network without a documented occupational exposure (10.5%). These findings support the concept that tuberculosis infection risk is determined by the interaction between exposure history, occupational environment, underlying disease, and the intensity and composition of immunosuppressive treatment rather than by any single risk factor.

The interpretation of these findings requires an important terminological and conceptual consideration. Current WHO guidance distinguishes *tuberculosis infection*, while acknowledging that available immunological tests detect an immune response to *M. tuberculosis* rather than viable bacilli. Neither tuberculin skin testing nor interferon-γ release assays (IGRAs) can by themselves distinguish tuberculosis infection from active tuberculosis disease or determine whether an individual will subsequently develop disease [10,11]. Therefore, in the present study, a positive immunological test in an asymptomatic individual without radiological evidence of active disease should be interpreted as immunological evidence consistent with tuberculosis infection, rather than as definitive proof of a persistent viable latent infection. This distinction is particularly relevant when applying these findings to immunocompromised populations.

The observed prevalence of immunological positivity among household contacts was notably high, reaching 41.4%, and was approximately twice that observed among individuals with occupational exposure (21.2%). Moreover, household contacts had a significantly higher frequency of positive tests than both tuberculosis-facility healthcare workers and healthcare workers in the general medical network. This finding is biologically plausible because household exposure may involve prolonged and repeated close contact with an infectious source, potentially resulting in a greater cumulative exposure dose than that experienced during many occupational encounters [12,13]. The finding is also consistent with the established importance of contact investigation as a strategy for identifying individuals at increased risk of tuberculosis infection and subsequent disease.

The relatively high prevalence among tuberculosis-facility healthcare workers (22.6%) further indicates that occupational exposure remains an important determinant of tuberculosis infection. Importantly, this prevalence was similar to that among individuals with occupational contact with a patient with tuberculosis (21.2%), whereas it was significantly higher than that among healthcare workers in the general medical network without documented exposure (10.5%). The absence of a statistically significant difference between tuberculosis-facility workers and individuals with occupational exposure may indicate that the occupational environment itself represents a relevant exposure category, irrespective of whether a specific index patient has been documented [14,15]. Conversely, the presence of immunological positivity among general healthcare workers without a recognized exposure should not necessarily be interpreted as evidence of test misclassification; undocumented community or occupational exposures, remote exposure, and limitations of exposure ascertainment may contribute to this finding.

The most striking observation was the high prevalence of immunological positivity among heart-transplant recipients, which reached 53.6%. This proportion was substantially higher than that observed in patients with rheumatic diseases receiving immunosuppressive therapy (13.9%) [16,17]. The difference is clinically relevant because solid-organ transplantation represents a state of profound and sustained immune suppression and is associated with an increased risk of tuberculosis, including extrapulmonary and disseminated disease. Contemporary recommendations therefore support systematic assessment for tuberculosis infection in transplant candidates and recipients. Previous evidence also indicates that treatment of tuberculosis infection in solid-organ transplant populations can substantially reduce the incidence of subsequent tuberculosis disease, although disease may still occur despite preventive treatment.

The difference between heart-transplant recipients and patients receiving immunosuppression for rheumatic disease may reflect both the intensity and duration of immunosuppression and the specific pharmacological mechanisms involved. In our exploratory logistic regression analysis, tacrolimus and everolimus had the largest positive coefficients among the immunosuppressive agents evaluated in the heart transplantation group, and bootstrap stability analysis showed high sign consistency for these coefficients (100% and 97.6%, respectively). Age was also identified as a consistently important feature (100% sign consistency), and decision-tree analysis identified everolimus (importance 0.706) and age (0.294) as the most important features. These findings should, however, be interpreted as exploratory associations rather than causal effects. The observational design, small number of transplant recipients, and substantial correlation between transplantation status and multidrug immunosuppressive regimens preclude attribution of an independent causal effect to any individual drug.

The biological plausibility of an association between immunosuppressive treatment and tuberculosis risk is nevertheless considerable [18,19]. Calcineurin inhibitors, mammalian target of rapamycin (mTOR) inhibitors, corticosteroids, and other immunosuppressive agents interfere with cellular immune pathways that are essential for controlling *M tuberculosis*. The importance of treatment-specific effects is also supported by evidence from immune-mediated inflammatory diseases. For example, anti-TNF therapy has been associated with an increased risk of tuberculosis reactivation, particularly when combined with other immunosuppressive agents. Recent evidence further suggests heterogeneity in tuberculosis risk between individual TNF inhibitors, reinforcing the principle that the immunosuppressive regimen should be considered as a multidimensional exposure rather than as a binary variable indicating whether immunosuppression is present [20,21].

In patients with rheumatic diseases, the prevalence of immunological positivity was 13.9%. In exploratory analyses, age showed 100% sign consistency, whereas bDMARD treatment showed 84.6% sign consistency and sex only 55.0%. The logistic regression coefficient for bDMARD treatment was negative in the present dataset, whereas the decision-tree model did not identify bDMARD treatment as an important feature. This apparent discrepancy illustrates the instability of variable-importance estimates in small datasets and highlights the difference between statistical association, predictive contribution, and biological causality. In particular, a negative coefficient should not be interpreted as evidence that biological therapy protects against tuberculosis infection. Patients receiving biological therapy may differ systematically from those receiving conventional treatment with respect to disease phenotype, previous screening, duration of treatment, healthcare utilisation, socioeconomic factors, or previous tuberculosis exposure.

The finding that pre-transplant patients had a positivity rate of 35.7%, compared with 53.6% among heart-transplant recipients, is also clinically informative [22]. Although the difference did not reach statistical significance, the direction of the association is consistent with the hypothesis that intensified immunosuppression after transplantation may alter the clinical and immunological profile of tuberculosis infection. At the same time, a positive test before transplantation provides an important opportunity for intervention because tuberculosis infection can be assessed before the initiation or intensification of immunosuppression [23,24]. Current transplant guidance emphasises screening early during transplant evaluation and consideration of preventive therapy when tuberculosis infection is identified.

An important aspect of our study was the application of machine-learning methods to risk stratification. Rather than attempting to replace immunological testing, we investigated whether routinely available clinical and epidemiological characteristics could provide additional information for identifying individuals more likely to have immunological evidence of tuberculosis infection. The use of logistic regression provided an interpretable reference model, whereas decision trees allowed non-linear relationships and interactions to be explored. An important aspect of our study was the application of exploratory machine-learning methods to risk stratification. Rather than attempting to replace immunological testing, we investigated whether routinely available demographic and treatment-related characteristics could provide additional information for identifying individuals more likely to have immunological evidence of tuberculosis infection. The use of logistic regression provided an interpretable reference model, whereas decision trees allowed non-linear relationships and interactions to be explored. The convergence of the two approaches in identifying treatment-related variables as important predictors in the heart transplantation group provides preliminary support for incorporating immunosuppressive treatment characteristics into future risk stratification. However, the two approaches did not converge in the rheumatic disease group: bDMARD treatment had a negative coefficient in logistic regression but zero importance in the decision tree, illustrating the difficulty of variable-importance estimation in small datasets.

Nevertheless, the machine-learning component should currently be regarded as hypothesis-generating rather than as a clinically deployable prediction model. Contemporary reporting standards for clinical prediction models recommend transparent reporting of model development, predictor definitions, handling of missing data, resampling procedures, discrimination, calibration, and external validation. The TRIPOD+AI framework specifically extends these requirements to regression and machine-learning models, while PROBAST+AI provides a framework for assessing methodological quality, risk of bias, and applicability. In the present study, the relatively small sample sizes within individual risk groups substantially restrict the complexity and reliability of predictive modelling.

Another important consideration is the potential effect of immunosuppression on the sensitivity and interpretation of immunological tests. Immunosuppressive treatment may attenuate T-cell-mediated responses and potentially result in false-negative or indeterminate immunological results [25]. Consequently, the observed prevalence of positive tests in patients receiving immunosuppression may underestimate the true burden of *Tbc*. This issue is particularly relevant to transplant recipients and patients receiving biologic or targeted immunomodulatory therapies. The interpretation of a negative immunological test in such populations should therefore be integrated with epidemiological history, previous exposure, clinical status, imaging, and the anticipated intensity of immunosuppression rather than considered definitive evidence against tuberculosis infection.

Our findings also have implications for clinical risk assessment. The substantial differences between risk groups suggest that a uniform screening strategy may not provide equivalent diagnostic yield across populations. Household contacts and transplant candidates or recipients appear to represent particularly important populations for systematic tuberculosis infection assessment. Similarly, tuberculosis-facility healthcare workers may benefit from structured occupational screening, particularly in settings with ongoing exposure to infectious and drug-resistant tuberculosis. For patients requiring immunosuppressive treatment, tuberculosis infection assessment should ideally be incorporated into the pre-treatment or pre-transplant evaluation, when clinically feasible.

The principal potential contribution of our approach is therefore not the replacement of conventional immunological testing, but the development of a multidimensional risk-stratification framework integrating exposure, demographic characteristics, underlying disease, and immunosuppressive treatment. Such an approach could eventually support prioritisation of patients for repeat testing, additional radiological assessment, specialist review, or preventive treatment. However, prospective validation is essential before such a model can be used to guide clinical decisions.

Although differences in LTBI prevalence were observed across several risk groups, the results of pairwise comparisons should be interpreted with caution. The relatively small sample sizes in certain subgroups, particularly healthy controls and pre-transplant participants, may have reduced statistical precision. Furthermore, multiple exploratory comparisons increase the likelihood of false-positive findings. Consequently, borderline statistical associations should be regarded as hypothesis-generating rather than confirmatory and require validation in larger independent cohorts.

## 5. Limitations

This study has several limitations that should be considered when interpreting the findings. The study was observational and predominantly cross-sectional. Consequently, the analysis identifies associations with immunological evidence of tuberculosis infection but cannot establish temporality or causal relationships between immunosuppressive treatment and tuberculosis infection. In particular, the coefficients observed for tacrolimus, everolimus, corticosteroids, and biological therapies should not be interpreted as causal treatment effects.

The number of participants differed substantially between risk groups, with particularly small numbers of patients undergoing pre-transplant assessment and heart-transplant recipients. This limited statistical power and resulted in wide confidence intervals for several prevalence estimates. The machine-learning models developed in these subgroups are therefore vulnerable to instability and overfitting.

The outcome used for modelling was the presence of a positive immunological test rather than incident active tuberculosis. This distinction is fundamental. A positive TST or IGRA provides evidence of an immune response to *M tuberculosis* but does not establish viable persistent infection and does not predict with certainty which individuals will develop active disease. Accordingly, the present models should be described as models of immunological evidence of tuberculosis infection, rather than validated models predicting progression from tuberculosis infection to active tuberculosis.

Model performance was estimated using repeated stratified 5-fold cross-validation (5 × 10 repeats) with nested hyperparameter tuning and pooled out-of-fold predictions. Bootstrap optimism correction (1000 resamples) was applied to estimate the residual optimism of ROC AUC, and 95% confidence intervals for all major metrics were obtained using stratified bootstrap. Calibration was assessed using the Brier score, calibration intercept, and calibration slope. However, external validation was not performed and remains essential before clinical implementation.

The number of events was small in both subgroups analysed with machine learning (9 of 71 in the rheumatic disease group and 19 of 41 in the heart transplantation group). This limits the complexity of models that can be reliably fitted and the precision of coefficient and feature-importance estimates. Bootstrap stability analysis showed that some coefficients were stable in sign (age in the rheumatic disease group, tacrolimus and everolimus in the heart transplantation group), whereas others were not (sex in the rheumatic disease group, mycophenolate mofetil in the heart transplantation group). Coefficients and feature-importance values should therefore not be interpreted as establishing a definitive hierarchy of causal risk factors.

Several epidemiological variables, including previous exposure to tuberculosis, type and intensity of contact, and bacterial shedding by the index case, were based on documented history. Misclassification of exposure is possible, particularly among healthcare workers without a recognized contact history. Undocumented community exposure may partly explain the presence of positive immunological tests among healthcare workers in the general medical network.

Immunosuppressive treatment was represented primarily by binary variables indicating exposure to individual drugs. This approach does not capture treatment duration, cumulative dose, treatment combinations, time since transplantation, trough concentrations, previous immunosuppressive regimens, or changes in therapy over time. These factors could materially influence tuberculosis risk and should be incorporated into future models.

The study was conducted within a defined healthcare setting and included participants from a limited geographic and epidemiological context. Differences in background tuberculosis incidence, BCG vaccination policies, prevalence of tuberculosis infection, occupational exposure, and patterns of immunosuppressive treatment may limit generalisability to other populations and healthcare systems.

External validation was not performed. In accordance with contemporary prediction-modelling standards, independent validation in geographically, clinically, and epidemiologically distinct cohorts is required before the proposed models can be considered for clinical implementation. TRIPOD+AI specifically emphasises transparent reporting of prediction-model development and evaluation, while PROBAST+AI highlights the importance of assessing risk of bias and applicability across participants, predictors, outcomes, and analytical methods.

An additional limitation concerns the statistical comparison strategy. Several subgroups included relatively few LTBI-positive participants, potentially limiting the reliability of asymptotic statistical tests and reducing statistical power. Moreover, the multiple pairwise comparisons were exploratory and were not adjusted for multiplicity. Therefore, the possibility of type I errors cannot be excluded, and findings with *p* values close to 0.05 should be interpreted cautiously.

## 6. Implications for Future Research

Future studies should therefore adopt a prospective, multicentre design with longitudinal follow-up to determine whether combinations of immunological, epidemiological, clinical, and treatment-related variables can predict incident active tuberculosis rather than merely immunological test positivity. Such studies should include serial IGRA/TBST measurements, detailed quantification of exposure, duration and intensity of immunosuppression, treatment combinations and cumulative doses, and systematic assessment of incident tuberculosis disease.

For the machine-learning component, future work should use external validation in geographically, clinically, and epidemiologically distinct cohorts before any clinical deployment. Model development should follow current reporting standards (TRIPOD+AI, PROBAST+AI), with pre-specified predictors and outcomes, appropriate handling of missing data, assessment of calibration in addition to discrimination, and confidence intervals for all major performance measures. Given the small number of events in the present subgroups (9 and 19), future models should be developed in substantially larger cohorts, ideally with a minimum of 10 events per candidate predictor, to ensure stable estimation of regression coefficients and feature-importance values. Pre-specified subgroup analyses should be performed separately for pre-transplant candidates and transplant recipients, since the present analysis was limited to a combined group.

## 7. Conclusions

This study demonstrates substantial heterogeneity in immunological evidence of *M tuberculosis* infection across clinically distinct risk groups, with particularly high positivity among heart-transplant recipients and individuals with household exposure to tuberculosis. The findings suggest that the risk profile of tuberculosis infection is influenced by both epidemiological exposure and the nature of immunosuppressive therapy. Exploratory machine-learning analyses further indicate that treatment-related variables may contribute to individualised risk stratification, particularly among transplant recipients and patients with rheumatic diseases. However, these findings should not be interpreted as evidence of causality or as a validated prediction of progression to active tuberculosis. Prospective multicentre studies with longitudinal follow-up and external validation are required to determine whether integrated clinical, epidemiological, immunological, and treatment-related models can reliably identify individuals at greatest risk of tuberculosis disease and thereby improve targeted prevention strategies.

## Figures and Tables

**Table 1:** Tuberculosis exposure among participants in high-risk groups.

High-Risk Group	Type of Tuberculosis Exposure	% (*n*)	95% CI
Health-care workers in specialised tuberculosis facilities (*n* = 93)	Occupational exposure, including exposure to patients with bacteriologically confirmed MDR tuberculosis	100.0 (93)	96.03–100.00
Health-care workers in general medical facilities (*n* = 68)	No documented contact with a patient with tuberculosis	0 (68)	0–5.35
Individuals with documented contact with a patient with tuberculosis (*n* = 95)	Household exposure	30.5 (29)	21.27–39.79
	Occupational exposure	69.5 (66)	60.21–78.73
	Exposure to a patient with bacteriologically confirmed tuberculosis	89.5 (85)	83.30–95.64
Patients with rheumatic diseases receiving immunosuppressive therapy (*n* = 79)	Occupational exposure	1.3 (1)	0.03–7.05
	No documented contact with a patient with tuberculosis	98.7 (78)	93.17–99.78
Patients undergoing heart transplantation and heart transplant recipients (*n* = 42)	No documented contact with a patient with tuberculosis	0 (42)	0.00–8.38

**Table 2:** Characteristics of immunosuppressive therapy in patients with rheumatic diseases.

Immunosuppressive Therapy	% (*n*)	95% CI
Biological disease-modifying antirheumatic drugs (bDMARDs)	83.5 (66)	75.37–91.72
—Tumour necrosis factor-α (TNF-α) inhibitors	48.5 (32)	36.43–60.54
—Interleukin-17A (IL-17A) inhibitors	15.2 (10)	6.50–23.80
—Interleukin-6 (IL-6) inhibitors	6.1 (4)	0.30–11.82
—Other bDMARDs	30.2 (20)	19.22–41.39
Conventional synthetic disease-modifying antirheumatic drugs (csDMARDs)	6.3 (5)	0.96–11.70
Incomplete treatment data	10.2 (8)	3.47–17.68

**Table 3:** Characteristics of patients undergoing pre-transplant assessment for heart transplantation.

Patient Characteristic	% (*n*)	95% CI
On the heart transplantation waiting list	7.1 (1)	1.27–31.47
Undergoing further assessment before heart transplantation	78.7 (11)	51.68–93.16
Voluntarily declined inclusion on the heart transplantation waiting list	7.1 (1)	1.27–31.47
Removed from the heart transplantation waiting list following multidisciplinary medical assessment	7.1 (1)	1.27–31.47

**Table 4:** Immunosuppressive drugs used in treatment regimens of heart transplant recipients (*n* = 28).

Immunosuppressive Drug	% (*n*)	95% CI
Tacrolimus	92.6 (26)	45.20–59.40 *
Everolimus	42.9 (12)	24.53–61.19
Mycophenolate mofetil	57.1 (16)	38.81–75.47
Ciclosporin	7.1 (2)	0.90–23.73
Glucocorticoids	32.1 (9)	14.84–49.44

* The percentage and 95% CI for tacrolimus should be verified against the original dataset before submission.

**Table 5:** Results of immunological testing in the study groups.

Study Group	Positive Results, % (*n*)	95% CI	Negative Results, % (*n*)	95% CI	χχ^2^	*p* Value
Patients with pulmonary tuberculosis	90.9 (111)	83.35–99.54	9.1 (11)	4.4–9.60	16.750	<0.0001 *
Health-care workers in specialised tuberculosis facilities (*n* = 93)	22.6 (21)	14.08–31.08	77.4 (72)	68.9–85.9	10.434	0.002 *
Health-care workers in general medical facilities (*n* = 68)	10.5 (7)	3.12–17.8	89.5 (60)	82.2–96.9	1.857	0.174
Individuals with household tuberculosis exposure (*n* = 29)	41.4 (12)	23.5–59.3	58.6 (17)	40.70–76.6	21.600	0.001 *
Individuals with occupational tuberculosis exposure (*n* = 66)	21.2 (14)	11.35–31.1	78.8 (52)	68.93–88.7	8.716	0.004 *
Patients with rheumatic diseases receiving immunosuppressive therapy (*n* = 79)	13.9 (11)	6.3–21.6	86.1 (68)	78.44–93.71	3.944	0.048 *
Patients undergoing pre-transplant assessment (*n* = 14)	35.7 (5)	16.18–61.40	64.3 (9)	38.60–83.8	13.147	0.001 *
Heart transplant recipients (*n* = 28)	53.6 (15)	35.81–70.47	46.4 (13)	29.53–64.2	28.106	0.001 *
Healthy controls (*n* = 69)	4.4 (3)	0.99–12.52	95.7 (66)	87.48–99.01	—	—

* Significant difference compared with the healthy control group.

**Table 6:** Pairwise comparisons of immunological test results between high-risk groups.

Group 1 vs. Group 2	χ^2^	*p* Value
Health-care workers in general medical facilities (*n* = 68) vs. individuals with household tuberculosis exposure (*n* = 29)	12.198	0.001 *
Health-care workers in general medical facilities (*n* = 68) vs. individuals with occupational tuberculosis exposure (*n* = 66)	2.897	0.089
Health-care workers in general medical facilities (*n* = 68) vs. health-care workers in specialised tuberculosis facilities (*n* = 93)	3.971	0.047 *
Health-care workers in specialised tuberculosis facilities (*n* = 93) vs. individuals with household tuberculosis exposure (*n* = 29)	3.959	0.047 *
Health-care workers in specialised tuberculosis facilities (*n* = 93) vs. individuals with occupational tuberculosis exposure (*n* = 66)	0.042	0.838
Individuals with household tuberculosis exposure (*n* = 29) vs. individuals with occupational tuberculosis exposure (*n* = 66)	4.122	0.043 *
Patients undergoing pre-transplant assessment (*n* = 14) vs. heart transplant recipients (*n* = 28) †	1.193	0.275
Patients with rheumatic diseases receiving immunosuppressive therapy (*n* = 79) vs. Heart transplant recipients (*n* = 28)	17.67	0.001 *

Note: * reliability of the obtained results at a p-value of less than 0.05.

**Table 7:** Exploratory machine-learning model performance in the two analysed subgroups.

Metric	Group Rheumatic Diseases, *n* = 71	Group Heart Transplantation, *n* = 41
Events/total	9/71	19/41
ROC AUC	0.670 (0.520–0.824)	0.668 (0.522–0.811)
PR-AUC	0.207 (0.083–0.393)	0.616 (0.420–0.827)
Bootstrap-corrected AUC	0.652	0.644
Sensitivity	0.522 (0.250–0.817)	0.579 (0.356–0.781)
Specificity	0.716 (0.613–0.814)	0.764 (0.596–0.905)
PPV	0.211 (0.071–0.384)	0.679 (0.451–0.876)
NPV	0.912 (0.830–0.977)	0.677 (0.498–0.857)
Cohen’s kappa	0.146 (−0.021 to 0.340)	0.346 (0.080–0.588)
MCC	0.171 (−0.028 to 0.382)	0.349 (0.082–0.599)
Brier score	0.238 (0.226–0.252)	0.241 (0.200–0.284)
Calibration intercept	0.133	0.467
Calibration slope	0.082	0.112
Optimal C (logistic regression)	1.0	0.3
Decision-tree hyperparameters	entropy, max_depth = 2, min_samples_leaf = 1, min_samples_split = 2	Gini, max_depth = 2, min_samples_leaf = 2, min_samples_split = 2

Note: All 95% confidence intervals were obtained using bootstrap resampling at the participant level (1000 resamples), preserving the clustering structure induced by repeated cross-validation. For each bootstrap iteration, participants were resampled with replacement, and all out-of-fold predictions from the same participant were retained together. This approach avoids pseudo-replication. The use of participant-level bootstrap (rather than naive bootstrap) increased the width of the 95% confidence intervals by a factor of approximately 2.6 for ROC AUC and 2.5–2.7 for PR-AUC. Bootstrap-corrected AUC values were obtained using optimism correction with 1000 resamples. Calibration intercept and slope were calculated on pooled out-of-fold predictions. Values for sensitivity, specificity, PPV, and NPV correspond to a classification threshold of 0.5.

## Data Availability

The data supporting the findings of this study are available from the corresponding author upon reasonable request, subject to applicable ethical and institutional restrictions.
